# Findings and significance of early follow-up biopsies after treatment of acute rejection in kidney transplant recipients

**DOI:** 10.3389/ti.2026.16296

**Published:** 2026-08-13

**Authors:** Amanda Ahlmark, Kaisa Ahopelto, Anne Räisänen-Sokolowski, Jouni Lauronen, Marko Lempinen, Ville Sallinen, Ilkka Helanterä

**Affiliations:** 1 Department of Transplantation and Liver Surgery, Helsinki University Hospital and University of Helsinki, Helsinki, Finland; 2 Department of Pathology, Helsinki University Hospital and University of Helsinki, Helsinki, Finland; 3 Finnish Red Cross Blood Service, Vantaa, Finland

**Keywords:** acute rejection, follow-up biopsies, histology, kidney transplantation, outcomes

## Abstract

Treatment resistant acute rejection after kidney transplantation is linked to inferior outcomes, yet many centers do not routinely perform follow-up biopsies. The optimal timing of such biopsies remains uncertain. We evaluated findings from follow-up biopsies performed ≤45 days after treatment of acute rejection and assessed their association with long-term outcomes. Adult kidney transplants performed from 01/2014 to 07/2024 were included (n = 2,420). Acute rejections were classified according to Banff criteria. T-cell–mediated rejection, including borderline TCMR changes, was treated with pulse steroids; antibody-mediated rejection was treated with steroids, intravenous immunoglobulin, and plasma exchange. Findings from follow-up biopsies taken ≤45 days after treatment of early acute rejection were correlated with graft outcomes. Of 383 patients (16%) with acute rejection within 3 months post-transplant, 221 underwent a follow-up biopsy ≤45 days. Of these, 50% were taken by protocol and 50% because of clinical reasons. In 164 cases (74%), rejection changes had resolved. At 1-year, median creatinine was 1.3 mg/dL in those without rejection, 1.6 mg/dL in patients with persistent changes, and 1.5 mg/dL in those with resolved changes. Persistent histologic rejection within 45 days was associated with inferior long-term graft survival.

## Graphical Abstract

**Figure:**
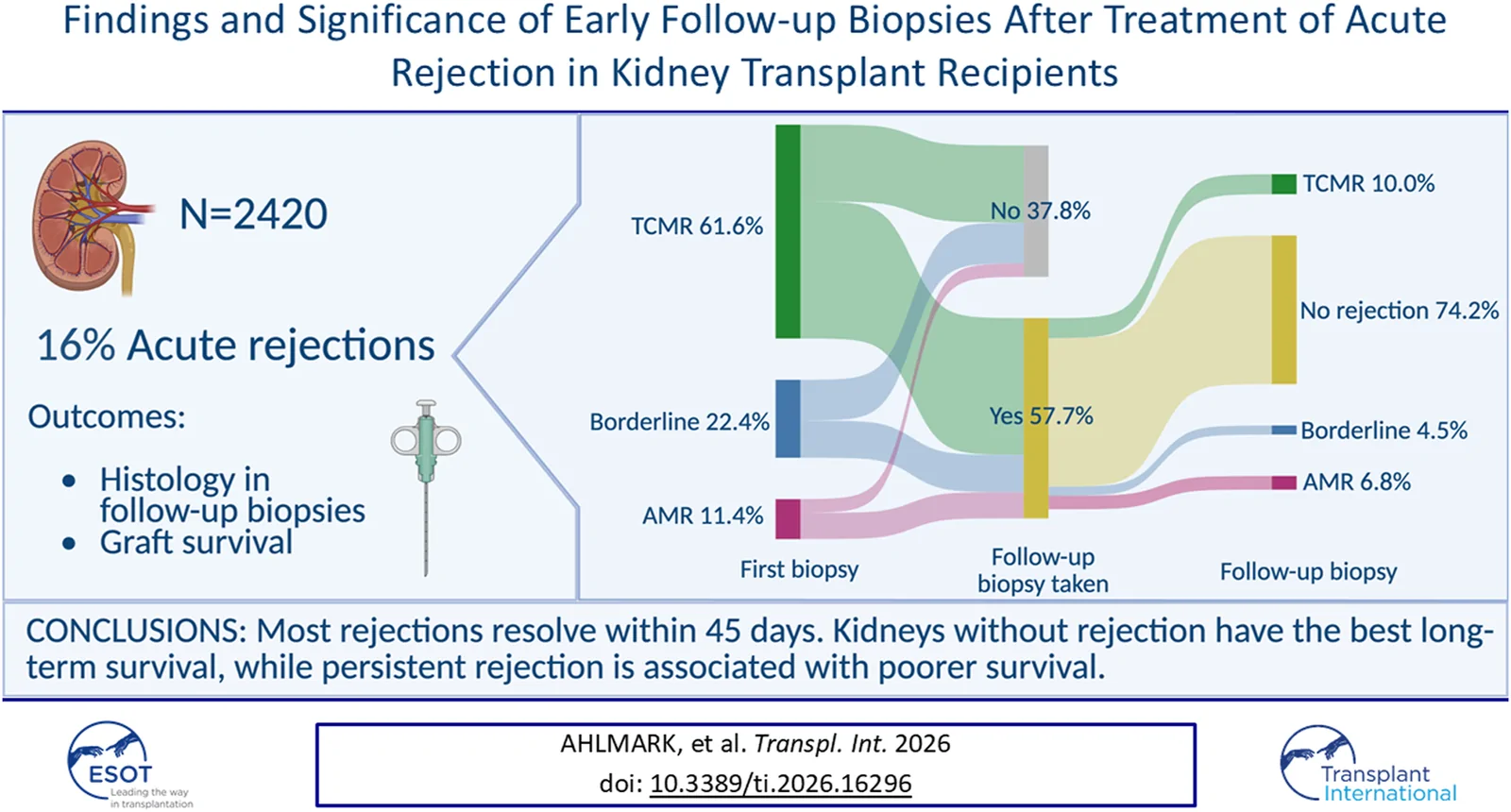

## Introduction

Up to 20% of patients experience rejection of the kidney allograft during the first weeks after transplantation [1–3]. The Banff classification of Transplant Pathology is used for reporting kidney transplant biopsies. Acute rejection is classified mainly into two different categories: T-cell-mediated rejection (TCMR), which is more common, and antibody-mediated rejection (AMR), which can be more difficult to treat [4]. Both types of rejection are associated with worse long-term graft survival [4–6]. Furthermore, borderline TCMRs are seen as a mild version of TCMR, but the views on the clinical significance of borderline TCMRs and their possible treatment differ [7, 8]. The Banff system was established in 1991 and has since undergone multiple updates, especially regarding AMR [2, 9].

To detect early rejection, a biopsy is recommended within the first few weeks post-transplantation in cases of early graft dysfunction or delayed graft function (DGF) [10]. Weekly biopsies have been recommended until kidney function is achieved, but later studies have shown that often one biopsy may be enough since the diagnosis seldom changes in subsequent biopsies [11, 12].

Follow-up biopsies after treating rejection are not always routinely taken but might offer valuable information to guide immunosuppressive therapy after rejection, helping clinicians to balance the need to detect persistent rejection early enough for effective intervention while avoiding unnecessary early biopsies that could lead to excessive immunosuppression.

There is no clear consensus for an optimal timing of a follow-up biopsy, although 3 months has been suggested [13].

At our institution, follow-up biopsies are usually taken 2–6 weeks post-treatment of acute rejection. We analyzed findings of these follow-up biopsies taken ≤45 days after treatment of acute biopsy-confirmed rejection and correlated the findings with long-term outcomes. We hypothesized that persistent rejection findings are associated with worse long-term outcomes.

## Methods and materials

### Study population and data collection

This is a retrospective observational registry analysis studying the results of follow-up biopsies after treatment of acute rejection and their association with long-term outcomes. The study population comprised all consecutive adult (>18 years) kidney transplantations performed at Helsinki University Hospital, Finland, from January 2014 to July 2024. All transplantations were complement dependent cytotoxicity crossmatch negative, examined with donor splenocytes between 2006 and 2015 and peripheral T- and B-cells thereafter. The donor pool consisted of living donors, brain-dead donors, and donors after circulatory death, and also included HLA-incompatible (when at least moderate donor specific antibodies existed pre-transplantation) and ABO-incompatible transplantations. The exclusion criteria were primary nonfunction and graft loss or death within 30 days after transplantation. Patients with missing data were excluded from relevant analyses.

Patient data were sourced from electronic medical records and the Finnish Kidney Transplant Registry, a legally mandated national registry for the follow-up of kidney transplant patients. Patients were followed until death, graft loss, or 31 December 2024.

One Lambda Labscreen® mixed and single antigen beads with Luminex® were used for pretransplant HLA antibody screening and identification using the HLA Fusion software (One Lambda Inc., Canoga Park, CA). DSAs were measured centrally. Donor HLA typing was performed in intermediate resolution, complemented with haplotype analyses. Median fluorescence intensity values over 1000 were considered positive. Posttransplant DSAs were not routinely monitored, but taken at rejection diagnostics at clinician’s discretion. The number of patients with pretransplant DSA are presented in Table 1. The individual MFI levels of all the DSAs were unfortunately not available for the purposes of this study.

**TABLE 1:** Characteristics of study population based on rejection occurrence.

Variable	Resolved rejection within 45 days^1^ N = 164	Persistent rejection within 45 days^1^ N = 47	No rejection^1^ N = 1988	p-value^2^
Recipient age (years)	54 (43.64)	55 (38.64)	57 (45.65)	0.16
Recipient sex	​	​	​	0.32
Female	57 (35%)	13 (28%)	744 (37%)	​
Male	107 (65%)	34 (72%)	1,244 (63%)	​
Previous kidney transplant	40 (24%)	10 (21%)	248 (12%)	<0.001*
Donor type	​	​	​	0.07
Living donor	13 (8%)	3 (6%)	245 (12%)	​
Brain-dead donor	149 (91%)	43 (91%)	1,659 (83%)	​
Circulatory death donor	2 (1%)	1 (2%)	84 (4%)	​
ABO-incompatible donation	0 (0%)	0 (0%)	29 (1%)	0.30
Donor sex	​	​	​	0.15
Female	89 (54%)	20 (43%)	931 (47%)	​
Male	75 (46%)	27 (57%)	1,057 (53%)	​
Donor age (years)	63 (52.68)	62 (45.70)	60 (49.67)	0.16
First biopsy diagnosis	​	​	​	0.04*
AMR	20 (12%)	8 (17%)	Not applicable	​
Borderline TCMR	37 (23%)	3 (6%)	Not applicable	​
TCMR	107 (65%)	36 (77%)	Not applicable	​
Delayed graft function	75 (46%)	20 (43%)	316 (16%)	<0.001*
Cold ischemia time (hours)	17 (12.21)	15 (11.18)	15 (9.19)	0.001*
DSAs present at time of transplantation^3^	27 (16%)	10 (21%)	0 (0%)	<0.001
Peak panel reactive antibodies >30%^4^	52 (32%)	19 (40%)	458 (23%)	0.002*
HLA AB mismatch	2 (2.3)	2 (2.3)	2 (2.3)	0.093
HLA DR mismatch	1 (1.1)	1 (1.1)	1 (0.1)	<0.001*
Follow up time (months)	63 (30.93)	52 (13.73)	54 (26.86)	0.09
Serum creatinine 1 year post transplantation mg/dL^5^	1.56 (1.28, 2.06)	1.61 (1.40.1.96)	1.30 (1.06.1.61)	<0.001*
Change in serum creatinine between rejection diagnosis and 1 year post transplantation mg/dL^6^	−2.15 (−4.6, −0.78)	−1.91 (−3.64, −1.1)	Not applicable	0.97

1 n (%); Median (IQR).

2 Fisher’s exact test; Pearson’s Chi-squared test; Kruskal-Wallis rank sum test.

Missing n.

3 891.

4 10.

5 821.

6 20.

*p-value statistically significant, <0.05.

AMR, antibody mediated rejection; TCMR, T-cell mediated rejection; DSAs, donor specific antibodies; HLA, human leukocyte antibodies.

Calculated panel reactive antibodies were calculated by comparing the known distribution of HLA antigens of Finns to the patient’s HLA antibody profile.

Delayed graft function was defined as the need for dialysis during the first post-transplant week.

This study was approved by the institutional review board of Helsinki University Hospital (HUS/23/2024). The clinical and research activities being reported are consistent with the Principal of Declaration of Istanbul as outlined in the Declaration of Istanbul on Organ Trafficking and Transplant Tourism.

### Immunosuppression and rejections

Corticosteroids were used as induction therapy for all kidney recipients. In addition, basiliximab or anti-thymocyte globulin was administered to patients with immunologic risk factors. Post-transplant maintenance immunosuppression comprised corticosteroids, mycophenolate, and a calcineurin inhibitor (tacrolimus or cyclosporine A). In 2014–2019, Tacrolimus was chosen in immunologically higher-risk patients and in all patients after 2019. From 2023 onwards, virtual crossmatch was done prior to kidney allocation on all patients, and donor-specific antibodies (DSA)-positive transplantations were generally avoided.

All biopsies were paraffin-embedded. Histopathologic changes described were scored by experienced renal pathologists using the Banff scoring system at the time, and the histopathological analyses were not reassessed for this study. TCMR, including borderline TCMR changes, were treated with pulse corticosteroids, and AMR typically with corticosteroids, intravenous immunoglobulin, and plasma exchange. Anti-thymocyte globulin was used in 10 cases to treat steroid resistant TCMR. Cases of probable AMR, which were treated as AMR, were defined as AMR for the purposes of this study.

### Statistical analyses

Borderline TCMR changes were considered rejection in the main analysis. In sensitivity analysis, borderline TCMR changes in follow-up biopsies after treated TCMR were considered as a resolved rejection. In addition, survival analyses were repeated excluding borderline changes from the rejection cohort.

Categorical variables are described as the number of cases and percentages. Continuous variables are described as median and interquartile range, as the distributions within the dataset were not normal. Kruskal-Wallis rank sum test was used to assess statistical significance for differences in the continuous variables, Fisher’s exact test was used for categorical data with small sample sizes, and Pearson’s Chi-squared test was used for categorical data with larger sample sizes. Histograms and Sankey diagrams were used to graphically describe the rates of acute rejection and follow-up biopsy findings.

Graft survival was analyzed using Kaplan-Meier survival curves with both death with functioning graft and graft loss as outcomes. Sensitivity analyses were done with only death-censored graft survival. Differences between the groups were analyzed with the log-rank test. Two-sided p-values <0.05 were considered statistically significant.

Risk factors for persistent rejection were studied using multivariable logistic regression analysis. Variables for the regression analysis were chosen based on known risk factors for acute rejection, as well as clinical interest. Graft function was not included in the main model due to possible confounding, but was included in supplemental models. Possible multicollinearity was studied using the variance inflation factor.

Patients with missing data were excluded from relevant analyses. In survival analyses, patients who died or lost their graft within 30 days of transplantation were excluded (n = 9).

Statistical analyses were performed using R (R Core Team, 2024), R Studio (Posit Software, 2025), and the R packages dplyr, tidyverse, readxl, ggplot2, patchwork, gtsummary, rms, broom, survival, survminer, and stats.

## Results

A total of 2,420 kidneys were transplanted during the study period. Patients with primary non-function (n = 49) and follow-up time <30 days after transplantation (n = 9), were excluded.

**FIGURE 1:**
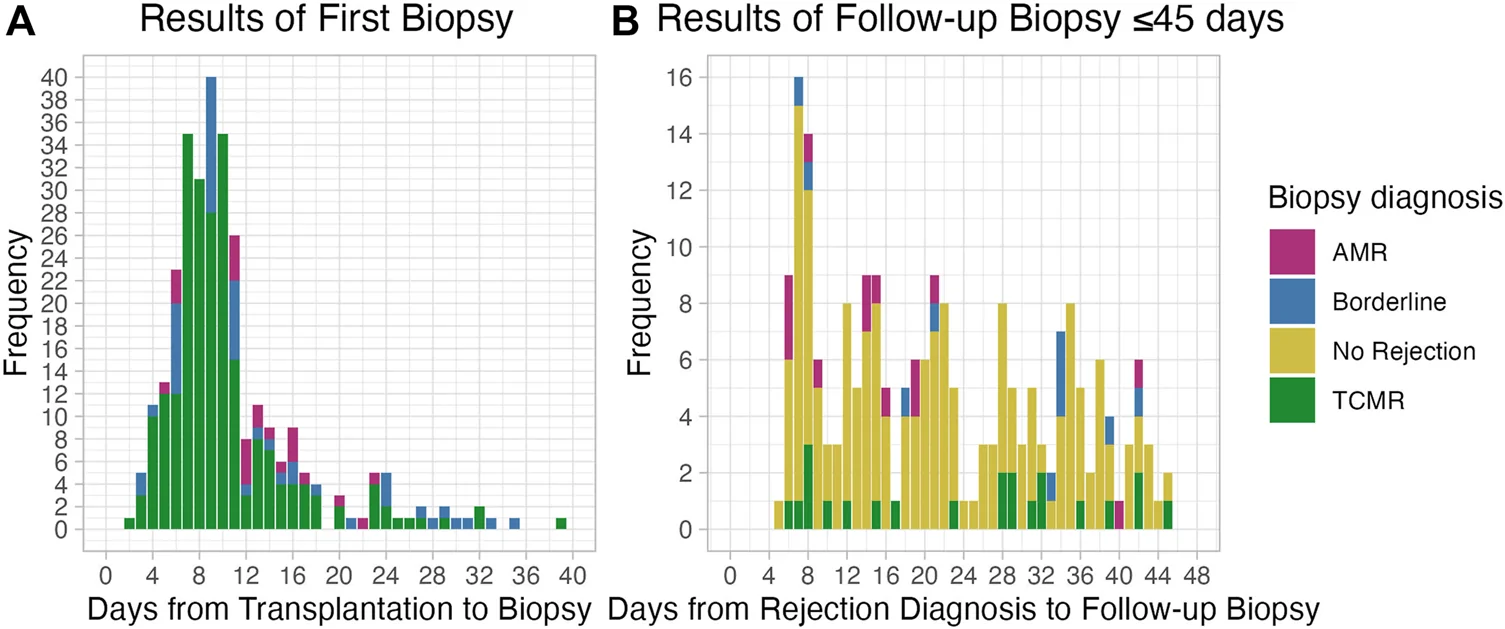
**(A,B)** Biopsy diagnoses of first biopsy **(A)** and results of follow-up biopsy ≤45 days **(B)**.

**FIGURE 2:**
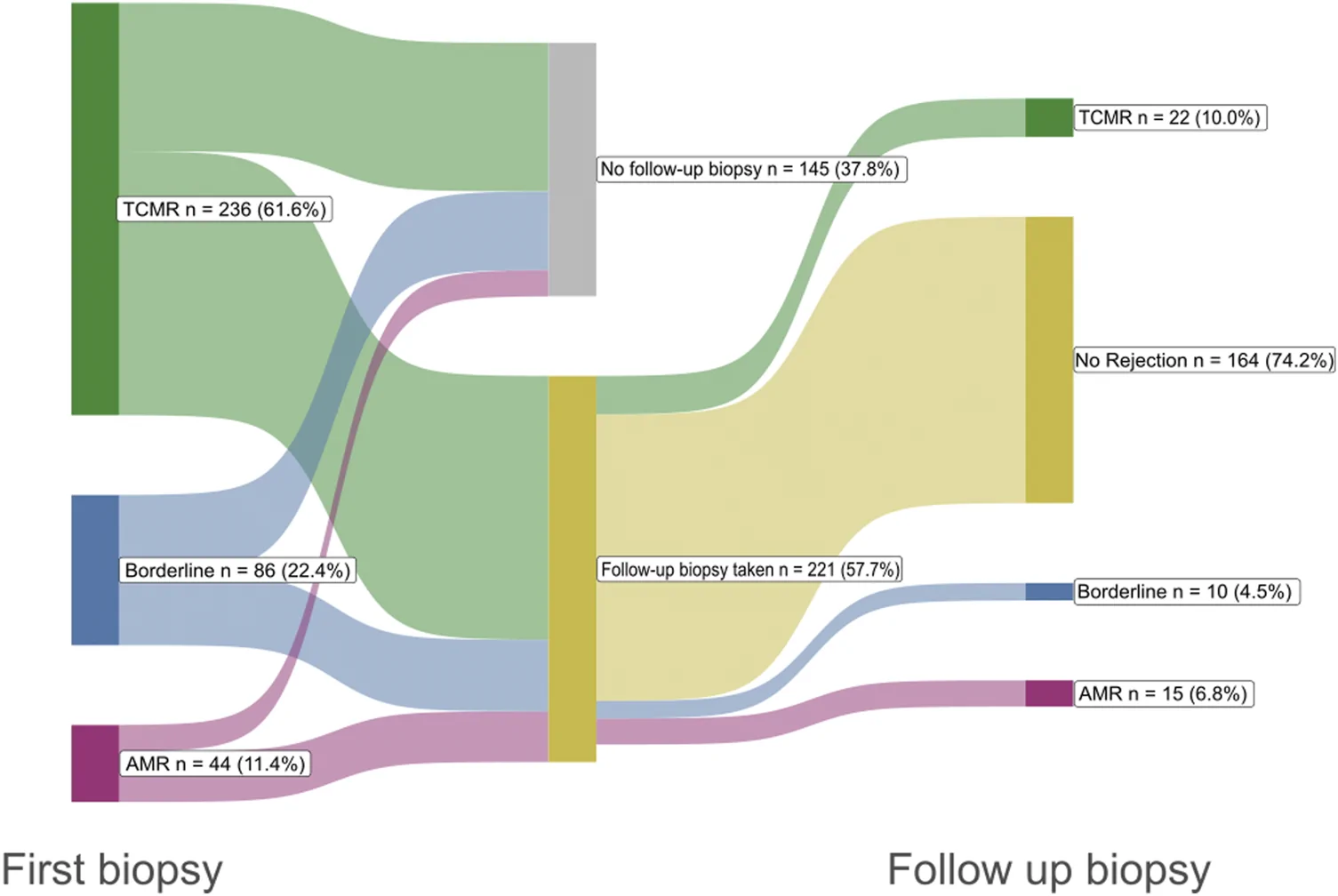
Sankey diagram on the evolution of biopsy diagnosis from rejection diagnosis to follow up.

Among 2,362 patients, 383 (16%) experienced acute rejection within the first 3 months post-transplant. Of these, 236 (61%) were TCMR, 44 (11%) AMR, and 86 (22%) borderline TCMR; 17 (4%) had missing detailed biopsy data. Within 45 days post-rejection, 221 (58%) of the patients with acute rejection had at least one follow-up biopsy taken. In 74% (n = 164), the rejection changes had already resolved (Figures 1A,B). Persisting rejection changes consisted of 6.8% AMR (n = 15), 4.5% borderline TCMR (n = 10), and 10.0% TCMR (n = 22). 4.5% (n = 10) of the follow-up biopsies were inadequate or unclear (Figure 2). In clinical practice, if TCMR was downgraded to borderline TCMR rejection in the follow-up biopsy, the rejection was considered resolved, and no new rejection therapy was started.

Basic characteristics of included patients are shown in Table 1. Patients with previous kidney transplantation had a higher risk of acute rejection during the first 3 months. The presence of DSAs at the time of transplantation and a peak panel reactive antibodies >30 was associated with acute rejection, with the highest rates observed in the group with persistent rejection changes within 45 days. Patients with acute rejection had a significantly higher creatinine at 1 year post-transplantation, with the highest levels in those with persistent rejection changes within 45 days, compared to those with no rejection. There was no clinically or statistically significant difference in serum creatinine change from initial rejection diagnosis to 1 year post transplant, between those who had resolved rejection and persistent rejection (Table 1).

**FIGURE 3:**
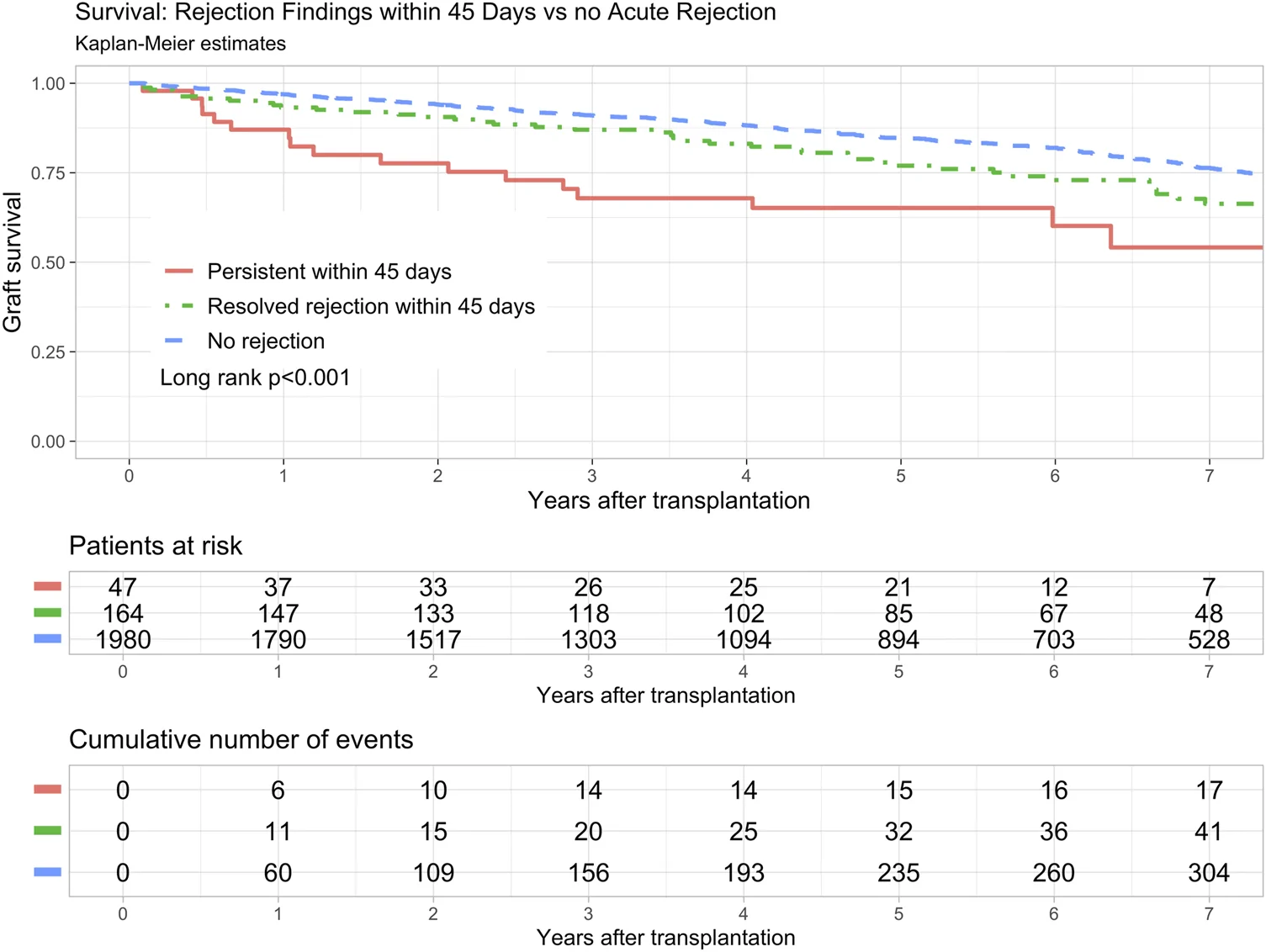
Survival estimates based on rejection findings within 45 days, graft survival.

In Kaplan-Meier survival estimates, the survival of patients with persistent rejection within 45 days was significantly worse than those who had resolved rejection or no acute rejection (Figure 3). The survival rates at 1, 3, and 7 years were 87%, 68%, and 54%, respectively, for patients with persistent rejection; 93%, 87%, and 66% for those with resolved rejection; and 97%, 91%, and 76% for those with no acute rejection. The best survival was observed in patients with no rejection changes during the first 3 months (p < 0.001, Figure 3). These trends could also be observed in death-censored survival analysis, with less prominent but still significant differences in survival (Supplementary Figure S1). Patients with no follow-up biopsy taken after treatment of acute rejection had similar survival as those with resolved rejection changes within 45 days (Supplementary Figure S2). The patient characteristics based on the availability of follow-up biopsy are presented in Supplementary Table S1. Follow-up biopsies were more commonly performed in patients with delayed graft function, whereas an initial diagnosis of borderline TCMR rejection less often led to a follow-up biopsy compared to AMR or TCMR.

In sensitivity analyses with borderline TCMR considered resolved if following TCMR, similar trends in survival were observed as in the main analyses, but the survival estimates of patients with persistent rejection within 45 days were more inferior (Supplementary Figures S3, S4). Furthermore, inferior survival was observed in all TCMR grades including Borderline TCMR with persistent rejection changes (Supplementary Figure S5).

The indication for the follow-up biopsy was available for 210 of the 221 patients with acute rejection. Of these biopsies, 50% were performed according to protocol, and 50% were performed for clinical indications, such as rising serum creatinine or failure of serum creatinine to decline. In Kaplan-Meier survival analyses, the survival of patients with clinical indications for follow-up biopsy had the worst survival (Supplementary Figures S6, S7).

In multivariable logistic regression analysis, patients with borderline TCMR rejection had a lower risk for persistent rejection findings within 45 days (OR 0.22, 95% CI 0.05–0.69, p = 0.03). No other risk factors were identified (Table 2). In supplemental analyses with graft function included no differences in risk could be observed (Supplementary Table S2). In a subgroup of patients with delayed graft function, there were no differences in risk (Supplementary Table S3). In a subgroup of patients with early function (Supplementary Table S4), male recipients and male donors were associated with a higher risk of persistent rejection. Patients with borderline TCMR rejection had a lower risk of persistent rejection. The risk was also lower for patients with a previous kidney transplant. Patients with previous transplants were generally younger in this subgroup.

**TABLE 2:** Multivariable logistic regression analysis of factors associated with persistent rejection within 45 days.

Characteristic	OR	95% CI	p-value
Recipient sex: Male	2.30	0.99, 5.88	0.054
Recipient age (years)	1.01	0.97, 1.05	0.6
Peak PRA over 30: Yes	1.90	0.69, 5.24	0.2
Donor sex: Male	1.50	0.73, 3.14	0.3
Donor age (years)	0.97	0.94, 1.01	0.11
Previous kidney transplant	0.43	0.13, 1.29	0.14
HLA AB mismatch	0.90	0.62, 1.33	0.6
HLA DR mismatch	0.84	0.42, 1.68	0.6
First rejection diagnosis	​	​	0.037*
TCMR	—	—	​
AMR	1.02	0.29, 3.49	​
Borderline TCMR	0.23	0.05, 0.74	​
Cold ischemia time (hours)	0.96	0.86, 0.99	0.03
Donor specific antibodies at time of transplantation	1.61	0.47, 5.52	0.4
Donor type	​	​	0.3
DBD	—	—	​
DCD	0.37	0.01, 4.65	​
LD	0.33	0.05, 1.61	​

*p-value statistically significant, <0.05.

PRA, panel reactive antibodies; HLA, human leukocyte antibodies; TCMR, T-cell mediated rejection; AMR, antibody mediated rejection; DBD, donation after brain-death; DCD, donation after circulatory determination of death; LD, living donor.

The rejection grades in the initial and follow-up biopsies are presented in Table 3. In this cohort, 26 cases presented with an isolated v-lesion. Of these, 5 were in recipients from living donors and 3 in recipients from DCD. The changes in lesion scores from baseline to follow-up biopsy are presented in Supplementary Table S5. Generally, lesion scores remained the same or increased in persisting rejections. Also the activity lesion, which was calculated as described by Vaulet et al [14], increased or remained the same more often in persisting rejections.

**TABLE 3:** Rejection grades.

Rejection type	First biopsy, N	First follow-up biopsy, N	Second follow-up biopsy, N	Third follow-up biopsy, N
AMR	44	18	6	4
Borderline TCMR	86	13	4	4
TCMR IA	60	8	1	NA
TCMR IB	12	2	2	NA
TCMR IIA	126	30	7	NA
TCMR IIB	38	6	2	1
No rejection	NA	168	47	13
Inadequate	NA	8	4	2

TCMR, T-cell mediated rejection; AMR, antibody mediated rejection.

## Discussion

In this retrospective study on the findings of follow-up biopsies after treating acute rejection in kidney transplantation, we found that most rejection findings were resolved within 45 days. There are no recent guidelines on when and if a follow-up biopsy after treated rejection should be taken. KDIGO guidelines on transplant recipient care in 2009 recommend a follow-up biopsy if creatinine does not return to baseline level after treated rejection [10]. However, treating remaining histological rejection changes, even in patients with recovered kidney function, might be beneficial for kidney allograft survival [15]. Following our study, we conclude that follow-up biopsies could be performed earlier than the suggested 3-month mark.

Rejection is common after kidney transplantation despite improvements in immunosuppressive medication over the past decades [16]. The rate varies across studies between 15 to over 20 percent [17]. The rejection rate in this study population, comprising all kidney transplantations at our institution over 10 years, is well in line with reports from other centers.

Paris Transplant Group reports that at a 3-month posttreatment biopsy, 13.3% of patients showed persisting TCMR changes [13]. In another study, 79% of patients had a complete response to rejection treatment histologically when treated with corticosteroids or with corticosteroids and ATG (17/163 patients) [15]. They gave further treatment to all but one of the patients (n = 34) with remaining histological rejection regardless of kidney function and reached complete response in an additional 27 patients. A Canadian study presented worse graft survival in persistent TCMR, when follow-up biopsies were taken within 6 months post-rejection treatment, with a study population having persisting or subsequent TCMR in 64% of cases [18]. In our study, where a follow-up biopsy was taken earlier, 10% of patients had persisting TCMR changes. Additionally 4.5% had borderline TCMR changes not warranting additional treatment. Thus, changes classified as TCMR had cleared earlier than previously reported. This opens an opportunity to intervene earlier to improve kidney function in more patients. Patients with persisting rejection within 45 days post-treatment had worse graft survival. We included patients who had rejection within the first three months post-transplantation, but most of the rejections were discovered at a median time of 9 days since transplantation.

In our study, we found that graft survival in those with rejection, and especially those with persistent rejection, is worse than in patients with no rejection. Persistent rejection changes are associated with the formation of fibrosis and loss of graft function, which essentially leads to graft loss in the long term [19]. In addition to risk of graft loss, acute rejections can also be associated with worse patient survival. Return to dialysis worsens patient outcomes by increasing the risk for both cardiovascular disease and cachexia [20, 21]. Immunosuppression increases the risk of cancer. A Finnish population registry study comprising over 6500 solid organ transplant recipients showed that heart transplant recipients are at the highest risk of developing cancer [22]. Heart transplant recipients receive higher doses of maintenance immunosuppression compared to kidney transplant recipients and often receive anti-thymocyte globulin/anti-human T-lymphocyte immunoglobulin induction. Immunosuppression also causes higher rates of infection, with around 50% of kidney transplant recipients experiencing some kind of infection during the first year after transplantation [23]. The higher immunosuppression doses and the adverse effects associated with these could also partly explain why those with persistent rejection after kidney transplantation have worse survival.

At our center, borderline TCMRs in a first biopsy are treated as TCMR, with corticosteroids. Practices for the treatment of borderline TCMR changes vary [24]. It has been suggested that treating subclinical borderline TCMR might not be beneficial, but treatment if the biopsy was taken on clinical indications (i.e., impaired kidney function) has been associated with improved kidney function [8]. In sensitivity analyses, we observed similar results, supporting the treatment of borderline TCMRs. On a similar note, the dichotomous view of rejections being either TCMR or AMR has been questioned. A recent review by Callemeyn et al provides insight into the current evidence on the serology, histology, and prognosis of different rejection phenotypes, highlighting the gap in the current dichotomous classification [25]. In our study population, 26 cases presented with an isolated v-lesion TCMR. Their number remains so low, that subgroup analyses were not deemed justified.

This study consists of a large number of kidney transplant recipients. There are some limitations to consider, the retrospective nature of the study being one of them. In Finland, the follow-up of kidney transplant recipients is done by nephrologists in local hospitals around the country. These hospitals are required to report kidney graft and patient data to the National Transplant Registry. However, this does not always include data on biopsies taken regionally. Furthermore, a follow-up biopsy was not taken in all patients. In survival analysis, patients with missing follow-up biopsy post-rejection had similar survival as those with resolved rejection. Half of the follow-up biopsies taken were by protocol, and the survival of these patients was similar to those with missing follow-up biopsy. If a persistent rejection was found in the biopsy taken by protocol, the survival was slightly worse than those with resolved rejection findings, but the number of cases was low in this group.

As the immunosuppression regimen did not include induction therapy in the majority of patients, these results may not apply to all kidney transplant patients. But on the other hand, despite the induction-free therapy, most rejection changes had resolved within 45 days.

In conclusion, in our current study, the majority of rejections are resolved within 45 days after rejection. Early follow-up biopsies could allow earlier intervention in case of persistent rejection. Long-term survival is significantly better in kidneys with no rejection, while persistent rejection is associated with worse outcomes.

## Data Availability

The datasets presented in this article are not readily available because The individual-level data is not available due to national regulations and laws in Finland regarding the sharing of individual-level patient data. Aggregate-level data can be shared based on a reasonable request. Requests to access the datasets should be directed to amanda.ahlmark@helsinki.fi.
