# Supplementary material for: Findings and significance of early follow-up biopsies after treatment of acute rejection in kidney transplant recipients
Source: Transpl Int. 2026 Aug 13;39:16296. doi: 10.3389/ti.2026.16296 (PMC13518296; doi:10.3389/ti.2026.16296)
Supplement: Supplementary file 1 [file DataSheet1.docx]

## Findings and Significance of Early Follow-Up Biopsies After Treatment of Acute Rejection in Kidney Transplant Recipients

**Capsule sentence:** Most acute rejection episodes resolve within 45 days. Long-term graft survival is best among patients with no rejection and worst in persistent rejection.

Supplemental material

**Supplemental Table 1:** Characteristics of patients with rejection based on follow-up biopsy occurrence

**Supplemental Table 2:** Multivariable logistic regression analysis of factors associated with persistent rejection within 45 days, including graft function

**Supplemental Table 3:** Multivariable logistic regression analysis of factors associated with persistent rejection within 45 days, only patients with delayed graft function

**Supplemental Table 4:** Multivariable logistic regression analysis of factors associated with persistent rejection within 45 days, only patients with early graft function

**Supplemental Table 5:** Changes in lesion scores between baseline and follow up biopsy based on persistence of rejection diagnosis

**Supplemental Figure 1:** Survival estimates based on rejection findings within 45 days, death-censored graft loss

**Supplemental Figure 2:** Survival estimates based on rejection findings within 45 days, including survival for those without follow-up biopsy post-treatment, treated acute rejection. Graft loss and death with a functioning graft

**Supplemental Figure 3:** Survival estimates, where Borderline TCMR findings following TCMR were considered resolved, graft loss, and death with functioning graft

**Supplemental Figure 4:** Survival estimates, where Borderline TCMR findings following TCMR were considered resolved, death-censored graft loss

**Supplemental Figure 5:** Survival estimates based on initial rejection diagnosis and findings of follow-up biopsy

**Supplemental Figure 6:** Survival estimates based on rejection findings at 45 days and indications for biopsy

**Supplemental Figure 7:** Survival estimates based on indications for biopsy

## Tables

**Supplemental Table 1:** Characteristics of patients with rejection based on follow-up biopsy occurrence

| **Supplemental Table 1:** Characteristics of patients with rejection based on follow-up biopsy occurrence | | | |
| --- | --- | --- | --- |
| **Variable** | **Follow-up biopsy taken**^1^ N = 221 (58%) | **No follow-up biopsy**^1^ N = 162 (42%) | **p-value**^2^ |
| **Recipient** **Age (years)** | 54 (43,64) | 56 (46,64) | 0.21 |
| **Recipient Sex** |  |  | 0.96 |
| Female | 74 (33%) | 53 (33%) |  |
| Male | 147 (67%) | 109 (67%) |  |
| **Previous Kidney Transplant** | 50 (23%) | 26 (26%) | 0.12 |
| **Donor sex** |  |  | 0.33 |
| Female | 116 (52%) | 94 (58%) |  |
| Male | 105 (48%) | 68 (42%) |  |
| **Donor age (years)** | 63 (51,68) | 60 (52,70) | 0.89 |
| **First Biopsy Diagnosis** |  |  | 0.02* |
| AMR | 29 (13%) | 15 (10%) |  |
| Borderline | 41 (19%) | 45 (31%) |  |
| TCMR | 151 (68%) | 85 (59%) |  |
| **Delayed Graft Function** | 101 (46%) | 38 (23%) | <0.001* |
| **Cold Ischemia Time (hours)** | 16 (11,20) | 14 (9,19) | 0.003 |
| **DSAs present at time of transplantation** | 39 (18%) | 20 (12%) | 0.2 |
| **Peak panel reactive antibodies >30%** | 73 (33%) | 43 (27%) | 0.21 |
| **HLA AB mismatch** | 2 (2,3) | 2 (2,3) | 0.035 |
| **HLA DR mismatch** | 1 (1,1) | 1 (1,1) | 0.54 |
| **Follow up time (months)** | 58 (28,84) | 38 (17,66) | <0.001* |
| **Serum creatinine 1 year post transplantation mg/dL** | 1.58 (1.31, 2.06) | 1.50 (1.21,1.80) | 0.055 |
| *^1^* n (%); Median (Q1,Q3)  *^2^* Fisher’s exact test; Pearson’s Chi-squared test; Kruskal-Wallis rank sum test | | | |

**Supplemental Table 2:** Multivariable logistic regression analysis of factors associated with persistent rejection within 45 days, including graft function

| **Supplemental Table 2:** Multivariable logistic regression analysis of factors associated with persistent rejection within 45 days | | | |
| --- | --- | --- | --- |
| **Characteristic** | **OR** | **95% CI** | **p-value** |
| **Recipient** **Age (years)** | 1.01 | 0.97, 1.05 | 0.6 |
| **Recipient Sex (male)** | 2.32 | 0.99, 5.95 | 0.053 |
| **Previous Kidney Transplant** | 0.43 | 0.13, 1.28 | 0.13 |
| **Donor sex (male)** | 1.52 | 0.73, 3.22 | 0.3 |
| **Donor age (years)** | 0.97 | 0.94, 1.01 | 0.12 |
| **First Biopsy Diagnosis** |  |  | 0.036* |
| TCMR | - | - |  |
| Borderline TCMR | 0.23 | 0.05, 0.73 |  |
| AMR | 1.00 | 0.28, 3.46 |  |
| **Delayed Graft Function** | 0.91 | 0.42, 1.98 | 0.8 |
| **Cold Ischemia Time (hours)** | 0.93 | 0.86,1.00 | 0.038* |
| **DSAs present at time of transplantation** | 1.64 | 0.47, 5.71 | 0.4 |
| **Peak panel reactive antibodies >30%** | 1.90 | 0.69, 5.24 | 0.2 |
| **HLA AB mismatch** | 0.90 | 0.62, 1.33 | 0.6 |
| **HLA DR mismatch** | 0.85 | 0.42, 1.69 | 0.6 |
| **Donor type** |  |  | 0.3 |
| DBD | - | - |  |
| DCD | 0.37 | 0.01, 4.73 |  |
| LD | 0.32 | 0.05, 1.59 |  |
| * p-value statistically significant, <0.05  CI = Confidence Interval, OR = Odds Ratio, TCMR = T-cell mediated rejection, AMR = antibody mediated rejection, HLA = human leukocyte antibody, LD = living donor, DCD = donation after circulatory determination of death, DBD = donation after brain-death | | | |

**Supplemental Table 3:** Multivariable logistic regression analysis of factors associated with persistent rejection within 45 days, only patients with delayed graft function

| **Supplemental Table 3:** Multivariable logistic regression analysis of factors associated with persistent rejection within 45 days, only patients with delayed graft function | | | |
| --- | --- | --- | --- |
| **Characteristic** | **OR** | **95% CI** | **p-value** |
| **Recipient** **Age (years)** | 0.98 | 0.91, 1.05 | 0.5 |
| **Recipient Sex (male)** | 1.3 | 0.39, 4.82 | 0.7 |
| **Previous Kidney Transplant** | 1.24 | 0.22, 6.35 | 0.8 |
| **Donor sex (male)** | 0.42 | 0.13, 1.29 | 0.13 |
| **Donor age (years)** | 1.03 | 0.96, 1.11 | 0.4 |
| **First Biopsy Diagnosis** |  |  | 0.5 |
| TCMR | - | - |  |
| AMR | 0.51 | 0.06, 3.39 |  |
| Borderline TCMR | 0.47 | 0.06, 2.30 |  |
| **Cold Ischemia Time (hours)** | 0.89 | 0.77, 1.00 | 0.05 |
| **DSAs present at time of transplantation** | 1.89 | 0.41, 8.95 | 0.4 |
| **Peak panel reactive antibodies >30%** | 1.49 | 0.34, 6.24 | 0.6 |
| **HLA AB mismatch** | 1.18 | 0.62, 2.27 | 0.6 |
| **HLA DR mismatch** | 1.17 | 0.36, 3.74 | 0.8 |
| CI = Confidence Interval, OR = Odds Ratio, TCMR = T-cell mediated rejection, AMR = antibody mediated rejection, HLA = human leukocyte antibody | | | |

**Supplemental Table 4:** Multivariable logistic regression analysis of factors associated with persistent rejection within 45 days, only patients with early graft function

| **Supplemental Table 4:** Multivariable logistic regression analysis of factors associated with persistent rejection within 45 days, only patients with early graft function | | | |
| --- | --- | --- | --- |
| **Characteristic** | **OR** | **95% CI** | **p-value** |
| **Recipient** **Age (years)** | 1.04 | 0.98, 1.10 | 0.2 |
| **Recipient Sex (male)** | 9.21 | 1.84, 68.7 | 0.005* |
| **Previous Kidney Transplant** | 0.09 | 0.01, 0.66 | 0.017* |
| **Donor sex (male)** | 4.61 | 1.53, 15.3 | 0.006* |
| **Donor age (years)** | 0.94 | 0.89, 0.99 | 0.009* |
| **First Biopsy Diagnosis** |  |  | 0.012* |
| TCMR | - | - |  |
| AMR | 2.54 | 0.27, 23.5 |  |
| Borderline TCMR | 0.08 | 0.00, 0.57 |  |
| **Cold Ischemia Time (hours)** | 0.96 | 0.86, 1.06 | 0.4 |
| **DSAs present at time of transplantation** | 1.08 | 0.09, 13.3 | >0.9 |
| **Peak panel reactive antibodies >30%** | 4.14 | 0.68,29.7 | 0.12 |
| **HLA AB mismatch** | 0.63 | 0.35, 1.12 | 0.12 |
| **HLA DR mismatch** | 0.79 | 0.27, 2.33 | 0.7 |
| **Donor type** |  |  | 0.9 |
| DBD | - | - |  |
| DCD | 0.62 | 0.01, 22.4 |  |
| LD | 0.58 | 0.07, 4.22 |  |
| * p-value statistically significant, <0.05  CI = Confidence Interval, OR = Odds Ratio, TCMR = T-cell mediated rejection, AMR = antibody mediated rejection, HLA = human leukocyte antibody, DSA = donor specific antibodies, LD = living donor, DCD = donation after circulatory determination of death, DBD = donation after brain-death | | | |

**Supplemental Table 5:** Changes in lesion scores between baseline and follow up biopsy based on persistence of rejection diagnosis

| **Supplemental Table 5:** Changes in lesion scores between baseline and follow up biopsy based on persistence of rejection diagnosis | | | |
| --- | --- | --- | --- |
| **Lesion change (follow‑up – baseline)** | **AR at 45 days**  N = 47*^1^* | **No AR at 45 days**  N = 164*^1^* | **p-value***^2^* |
| **Δ i (interstitial inflammation)** |  |  | 0.462 |
| -3 | 0 (0%) | 5 (3.1%) |  |
| -2 | 8 (18%) | 35 (22%) |  |
| -1 | 16 (36%) | 62 (39%) |  |
| 0 | 15 (33%) | 47 (29%) |  |
| 1 | 6 (13%) | 9 (5.6%) |  |
| 2 | 0 (0%) | 2 (1.3%) |  |
| Unknown | 2 | 4 |  |
| **Δ t (tubulitis)** |  |  | 0.023 |
| -3 | 0 (0%) | 3 (1.9%) |  |
| -2 | 2 (4.5%) | 19 (12%) |  |
| -1 | 13 (30%) | 60 (37%) |  |
| 0 | 24 (55%) | 78 (48%) |  |
| 1 | 4 (9.1%) | 2 (1.2%) |  |
| 2 | 1 (2.3%) | 0 (0%) |  |
| Unknown | 3 | 2 |  |
| **Δ v (intimal arteritis)** |  |  | 0.003 |
| -3 | 1 (2.2%) | 0 (0%) |  |
| -2 | 2 (4.3%) | 13 (8.1%) |  |
| -1 | 5 (11%) | 51 (32%) |  |
| 0 | 32 (70%) | 89 (56%) |  |
| 1 | 5 (11%) | 6 (3.8%) |  |
| 2 | 1 (2.2%) | 1 (0.6%) |  |
| Unknown | 1 | 4 |  |
| **Δ ptc (peritubular capillaritis)** |  |  | 0.131 |
| -2 | 2 (4.3%) | 6 (3.7%) |  |
| -1 | 4 (8.7%) | 24 (15%) |  |
| 0 | 30 (65%) | 116 (72%) |  |
| 1 | 7 (15%) | 12 (7.5%) |  |
| 2 | 3 (6.5%) | 2 (1.2%) |  |
| 3 | 0 (0%) | 1 (0.6%) |  |
| Unknown | 1 | 3 |  |
| **Δ C4d** |  |  | 0.224 |
| -4 | 1 (2.1%) | 1 (0.6%) |  |
| -3 | 1 (2.1%) | 5 (3.2%) |  |
| -2 | 0 (0%) | 3 (1.9%) |  |
| -1 | 3 (6.4%) | 8 (5.1%) |  |
| 0 | 40 (85%) | 133 (85%) |  |
| 1 | 0 (0%) | 5 (3.2%) |  |
| 2 | 2 (4.3%) | 0 (0%) |  |
| 3 | 0 (0%) | 1 (0.6%) |  |
| Unknown | 0 | 8 |  |
| **Δ g (glomerulitis)** |  |  | 0.363 |
| -3 | 2 (4.3%) | 2 (1.3%) |  |
| -2 | 2 (4.3%) | 10 (6.3%) |  |
| -1 | 2 (4.3%) | 13 (8.2%) |  |
| 0 | 35 (76%) | 124 (78%) |  |
| 1 | 3 (6.5%) | 5 (3.2%) |  |
| 2 | 0 (0%) | 2 (1.3%) |  |
| 3 | 2 (4.3%) | 2 (1.3%) |  |
| Unknown | 1 | 6 |  |
| **Δ activity lesion (baseline – follow-up), presented as median (IQR)** | -1 (-3, 0) | -2 (-4, -1) | 0.003 |
| **Δ activity lesion (baseline – follow-up), presented as N (%)** |  |  |  |
| Improved | 27 (61%) | 131 (87%) |  |
| No change | 9 (20%) | 9 (6%) |  |
| Worsened | 8 (18%) | 10 (6.7%) |  |
| Unknown | 3 | 14 |  |
| *^1^*n (%), median (IQR)  *^2^*Fisher's exact test | | | |

## Figures

**Supplemental Figure 1:** Survival estimates based on rejection findings within 45 days, death-censored graft survival

**Supplemental Figure 2:** Survival estimates based on rejection findings within 45 days, including graft survival for those without follow-up biopsy post-treatment, treated for acute rejection

**Supplemental Figure 3:** Survival estimates, where Borderline findings following TCMR were considered resolved, graft survival

**Supplemental Figure 4:** Survival estimates, where Borderline findings following TCMR were considered resolved, death-censored graft survival

**Supplemental Figure 5:** Survival estimates based on initial rejection grade and findings of follow-up biopsy

AMR = antibody mediated rejection, Borderline = Borderline T-cell mediated rejection, TCMR = T-cell mediated rejection

**Supplemental Figure 6:** Survival estimates based on rejection findings at 45 days and indications for biopsy

AR = acute rejection

**Supplemental Figure 7:** Survival estimates based on indications for biopsy
